# A Riemannian Revisiting of Structure–Function Mapping Based on Eigenmodes

**DOI:** 10.3389/fnimg.2022.850266

**Published:** 2022-05-25

**Authors:** Samuel Deslauriers-Gauthier, Mauro Zucchelli, Hiba Laghrissi, Rachid Deriche

**Affiliations:** Centre Inria d'Université Côte d'Azur, Valbonne, France

**Keywords:** brain structure-function mapping, functional connectivity, structural connectivity, eigenvalue decomposition, Riemannian distance

## Abstract

Understanding the link between brain structure and function may not only improve our knowledge of brain organization, but also lead to better quantification of pathology. To quantify this link, recent studies have attempted to predict the brain's functional connectivity from its structural connectivity. However, functional connectivity matrices live in the Riemannian manifold of the symmetric positive definite space and a specific attention must be paid to operate on this appropriate space. In this work we investigated the implications of using a distance based on an affine invariant Riemannian metric in the context of structure–function mapping. Specifically, we revisit previously proposed structure–function mappings based on eigendecomposition and test them on 100 healthy subjects from the Human Connectome Project using this adapted notion of distance. First, we show that using this Riemannian distance significantly alters the notion of similarity between subjects from a functional point of view. We also show that using this distance improves the correlation between the structural and functional similarity of different subjects. Finally, by using a distance appropriate to this manifold, we demonstrate the importance of mapping function from structure under the Riemannian manifold and show in particular that it is possible to outperform the group average and the so–called glass ceiling on the performance of mappings based on eigenmodes.

## 1. Introduction

Current imaging technology allows us to observe the brain's connectivity both from a functional and structural point of view, for example using resting state functional magnetic resonance imaging (MRI) and diffusion MRI tractography, respectively. These two views are not decoupled and provide complementary information of the connectivity, and more general architecture, of the brain. Indeed, a current representation of the brain is that of a network, where the white matter substrates allows communication between distant brain regions (Sporns et al., 2005). Given this perspective, it is natural to try to understand and quantify the link between brain structure and function. One strategy to quantify this link is to predict, at least partially, the brain's function from its structure, so called structure–function mapping. In addition to the overarching goal of understanding the brain's organization, the ability to predict the brain's function from its structure has the potential to improve our understanding and quantification of brain pathologies (Wang et al., 2017).

Recently, many different strategies have been proposed to predict functional brain connectivity from structural connectivity. These range from sophisticated biophysical models (Galán, 2008; Honey et al., 2009; Deco et al., 2011; Deco and Jirsa, 2012; Messé et al., 2015a,b) to simpler approaches that do not attempt direct physical modeling (Deligianni et al., 2013; Abdelnour et al., 2014, 2018; Meier et al., 2016; Saggio et al., 2016; Liang and Wang, 2017; Becker et al., 2018; Nozari et al., 2020; Benkarim et al., 2021)'. A subset of these models specifically rely on the eigendecomposition of the structural connectivity matrix or its Laplacian (Robinson et al., 2016; Preti and Van De Ville, 2019) with promising applications to pathology (Raj et al., 2012; Abdelnour et al., 2021). Focusing on this type of approach, our group recently showed that these mappings can be unified under a general formulation (Deslauriers-Gauthier et al., 2020), highlighting the relation between series expansion and eigenmode approaches (Tewarie et al., 2020). This allowed us to highlight the link between the different models and directly compare them. While some models achieved high prediction accuracy, we found that all current model based on the eigendecomposition were outperformed by a simple group average. A possible explanation for this result is that the variability of the structural and functional connectivity is not sufficient in healthy subjects to be captured by the models (Deslauriers-Gauthier et al., 2020). A second possibility, the focus of this work, is that the tools used to train the models and evaluate their performance, i.e., the Frobenius norm and Pearson correlation, are not adapted to the space of functional connectivity matrices. By adapting the notion of distance to the manifold of functional connectivity matrices, it may be possible to improve the performance of existing structure–function mappings.

In this work, we revisit existing structure–function mapping models based on eigenmodes from a Riemannian perspective. Instead of computing distances between functional matrices in the vector space of symmetric matrices, we instead propose to use an affine invariant metric inducing a Riemannian distance. In addition to altering the objective function used to identify the parameters of the different mappings, this also changes the benchmark used to evaluate them. In our previous work (Deslauriers-Gauthier et al., 2020), we showed that a simple group average, which was used as a reference model, outperformed all eigenmode models. However, the use of a Riemannian distance for positive define matrices leads to a unique and well defined Fréchet mean (You and Park, 2021) which we propose as a more appropriate reference over the direct mean used previously. With this change of perspective, we demonstrate the importance of mapping function from structure under the Riemannian manifold, we improve the prediction of functional connectivity from structural connectivity, and we overcome the performance of the group average and the so–called glass ceiling on the performance of mappings based on eigenmodes.

## 2. Materials and Methods

### 2.1. Structure–Function Mapping

The objective of structure–function mapping is to identify a function *f* that minimizes the functional


(1) $$\begin{array}{r} L\left(f\right)=\frac{1}{K}\overset{K}{\underset{k=1}{\sum }}d^{2}\left(f\left(S_{k}\right),F_{k}\right) \end{array}$$

where $S_{k}\in {\mathbb{R}}^{M\times M}$ and $F_{k}\in {\mathbb{R}}^{M\times M}$ are the structural and functional matrices of the *k*^th^ subject and *M* is the number of considered brain regions. The function *f*:ℝ^*M* × *M*^ → ℝ^*M* × *M*^ is the so called structure–function mapping whose parameters are optimized by minimizing Equation (1). Note that *f* could be defined indendently for each subject, but we focus here on the situation where a single mapping is optimized across all subjects. The function *d*(*A, B*) measures the distance between matrices *A* and *B*. If the Frobenius norm is used, Equation (1) reduces to the usual formulation of the problem which is to minimize


(2) $$\begin{array}{r} L\left(f\right)=\frac{1}{K}\overset{K}{\underset{k=1}{\sum }}||f\left(S_{k}\right)-F_{k}|{|}_{F}^{2}. \end{array}$$

Once the parameters of the mapping *f* minimizing Equation (1) have been identified, its performance can be assessed on a new set of subjects by evaluating


(3) $$\begin{array}{r} E\left(f\right)=\frac{1}{K^{\prime }-K}\overset{N}{\underset{k=K+1}{\sum }}d_{t}^{2}\left(f\left(S_{k}\right),F_{k}\right) \end{array}$$

where *d*_*t*_ is the testing function. Interestingly, while the Frobenius norm or Euclidean distance is sometimes used, a more common choice is to use the Pearson correlation (Becker et al., 2018). In this case, the parameters of the mapping identified may be suboptimal as the optimization criterion differs for the evaluation criterion. However, optimizing the correlation directly is challenging as it is scale invariant and thus does not have a unique solution. In addition, the correlation is not a metric as it does not satisfy the triangle inequality which may lead to unintuitive comparison results. A metric can be calculated from the correlation *via* its relation to the cosine similarity, but there is no intrinsic justification for the use of angular distance between functional matrices. When the Frobenius norm is used for both optimization and evaluation, the model parameters are optimal, but the notion of distance is not adapted to the manifold of functional connectivity matrices as we discuss in the following section.

### 2.2. Riemannian Structure–Function Mapping

In the context of structure–function mapping, the functional connectivity matrices are typically computed as the Pearson correlation between the resting state BOLD fMRI signal of different brain areas. Let *D*∈ℝ^*M* × *T*^ be the data matrix where each row contains the demeaned and normalized time series of a brain area. The functional matrix can be written as *F* = *DD*^*T*^ whose singular value decomposition is *UΣ*^2^*U*^*T*^. Functional connectivity matrices are therefore symmetric positive semidefinite. In practice, because the time series of a brain area is unlikely to be expressed exactly as a linear combination of the other areas, they are in fact symmetric positive definite (SPD). The space of SPD matrices is a convex smooth manifold of the Euclidean space, a convex half–cone in the vector space of symmetric matrices. Because of this conical structure, it is natural to consider Riemannian metrics on the space of SPD matrices. In particular, affine–invariant metrics have been suggested and induce a Riemannian distance


(4) $$\begin{array}{r} d^{2}\left(A,B\right)=\text{Tr}\left(\log^{2}\left(A^{-1/2}BA^{-1/2}\right)\right) \end{array}$$

where log is the matrix logarithm and Tr is the trace. These notions have been used in the context of brain connectivity to classify connectivity matrices (Dodero et al., 2015) and regression (Wong et al., 2018). The Riemannian nature of the problem has also been observed by Benkarim et al. (2021), although they rely on the Euclidean distance for optimization and the correlation for evaluation. You and Park (2021) also re–visited Riemannian geometry for functional connectivity matrices, but they did not consider structure–function mapping specifically. Using a Riemannian distance over the Euclidean distance or correlation has several advantages. First, the distance is not computed elementwise but rather considers the general structure of the matrix (You and Park, 2021). This is consistent with the notion that the structure–function relationship is not one to one (Mišić et al., 2016) and instead operates on a network level (Atasoy et al., 2016; Xie et al., 2021). Second, matrices with non-positive or infinite eigenvalues are both infinitely far from any SPD matrices (Lenglet et al., 2006; Pennec et al., 2006), capturing the notion that they do not correspond to valid functional connectivity matrices. Finally, this space is smooth and allows the computation of a unique geodesic, and thus a unique minimal distance, between any pair of functional connectivity matrices. We thus propose to revisit the structure–function mappings based on eigenmodes using the Riemannian distance of Equation (4).

### 2.3. Reference Model

In our previous work (Deslauriers-Gauthier et al., 2020), we argued that the performance of structure–function mappings should be compared to a suitable reference model. A natural benchmark is the Fréchet mean $\bar{F}$ which minimizes $\underset{k}{\sum }d^{2}\left(\bar{F},F_{k}\right)$ and can thus be seen as a zeroth order mapping, one that does not make use of structural information. When the Euclidean norm is used to estimate model parameters, it can be computed as the average of the functional matrices of the training set ${\bar{F}}_{E}=\frac{1}{K}\overset{K}{\underset{k=1}{\sum }}F_{k}$. However, when the distance is given by Equation (4), the Fréchet mean cannot be computed directly. It must instead be estimated using the following iterative scheme


(5) $$\begin{array}{r} {\bar{F}}_{t+1}={\bar{F}}_{t}^{1/2}\exp\left(\frac{1}{K}\overset{K}{\underset{k=1}{\sum }}\log\left({\bar{F}}_{t}^{-1/2}F_{k}{\bar{F}}_{t}^{-1/2}\right)\right){\bar{F}}_{t}^{1/2} \end{array}$$

which coverages to the unique value ${\bar{F}}_{R}$ minimizing $\underset{k}{\sum }d^{2}\left(\bar{F},F_{k}\right)$ (Pennec et al., 2006). We use the definition above as a the reference model for our proposed methodology.

### 2.4. Optimization Procedure

As was shown in our previous work (Deslauriers-Gauthier et al., 2020), all existing mappings based on eigenmodes can be written as


(6) $$\begin{array}{r} f\left(S\right)=\overset{N-1}{\underset{n=0}{\sum }}g\left(\lambda_{n}\right)h\left(u_{n}\right)+C \end{array}$$

where *C*∈ℝ^*N* × *N*^ is a constant symmetric matrix and where λ_*n*_∈ℝ and $u_{n}\in {\mathbb{R}}^{N}$ are the *n*th eigenvalue and eigenvector of *S*, respectively. For example, to reproduce the spectral mapping of Becker et al. (2018), we can define


(7) $$\begin{array}{r} g\left(\lambda \right)=\overset{P}{\underset{m=0}{\sum }}a_{p}\lambda^{p}\text{and}h\left(u_{n}\right)=Ru_{n}u_{n}^{T}R^{T} \end{array}$$

where *a*_*m*_∈ℝ and *R* is a rotation matrix. The formulation in Equation (6) allows us to devise a general optimization scheme for all existing mappings. The objective is to minimize Equation (1) where the distance is given by Equation (4), that is


(8) $$\begin{array}{r} L\left(f\right)=\frac{1}{K}\overset{K}{\underset{k=1}{\sum }}\text{Tr}\left(\log^{2}\left(F_{k}^{-1/2}f\left(S_{k}\right)F_{k}^{-1/2}\right)\right) \end{array}$$

By substituting the general form in Equation (6), we obtain


$$\begin{array}{ll} L\left(f\right) & =\frac{1}{K}\overset{K}{\underset{k=1}{\sum }}\text{Tr}\left(\log^{2}\left(F_{k}^{-1/2}\left(\overset{N-1}{\underset{n=0}{\sum }}g\left(\lambda_{n}\right)h\left(u_{n}\right)+C\right)F_{k}^{-1/2}\right)\right)\\ =\frac{1}{K}\overset{K}{\underset{k=1}{\sum }}\text{Tr}\left(\log^{2}\left(\overset{N-1}{\underset{n=0}{\sum }}g\left(\lambda_{n}\right)h_{k}^{\prime }\left(u_{n}\right)+C_{k}^{\prime }\right)\right) \end{array}$$

where


(9) $$\begin{array}{r} h_{k}^{\prime }=F_{k}^{-1/2}h\left(u_{n}\right)F_{k}^{-1/2}\text{and}C_{k}^{\prime }=F_{k}^{-1/2}CF_{k}^{-1/2}. \end{array}$$

To minimize this functional, we iteratively minimize over the parameters of each term *g*, *h*′, and *C*′ while keeping the others fixed in a manner similar to the coordinate descent algorithm. For the eigenmode weights *g*, the parameters were optimized using the Broyden–Fletcher–Goldfarb–Shanno (BFGS) algorithm *via* scipy (Virtanen et al., 2020). For *h*′ and *C*′, the parameters were optimized using Pymanopt^1^ to ensure the parameters remained on the manifold of rotation and symmetric matrices, respectively. In practice, the performance of all models converged after approximately three iterations.

### 2.5. Evaluation

We evaluated the performance of the mappings proposed by Abdelnour et al. (2014, 2018) and spectral mappings (Meier et al., 2016; Becker et al., 2018; Deslauriers-Gauthier et al., 2020) which were all implemented using the general model in Equation (6). The specific form of the mappings is provided in Table 1. For the specific cases of the diffusion mappings, the Laplacian of the structural matrix, defined as *L* = *I*−*D*^−1/2^*SD*^−1/2^ where *D* is the diagonal degree matrix, was used as input. For models relying on spectral mapping, the maximum order was set to *M* = 6. For each mapping, the parameters were learned using a leave-one-out cross-validation strategy, meaning *K*′ = 100 and *K* = 99 in Equation (3). The performance of each mapping was then evaluated by measuring the distance between the predicted and observed functional connectivity matrices using Equation (4) on the left out subject. Finally, the performance of Fréchet mean in Equation (5) was evaluated using the same strategy and used as a benchmark.

**Table 1:** Definition of the structure–function mappings based on eigenmodes and their number of degrees of freedom.

**References**	**Model**	**Degrees of freedom**
Abdelnour et al. (2014)	*F*(*L*) = *e*^−β*Lt*^	1
Abdelnour et al. (2018)	*F*(*L*) = *ae*^−α*L*^+*bI*	3
Meier et al. (2016)	$F\left(S\right)=\overset{M}{\underset{m=0}{\sum }}a_{n}S^{m}$	*M*+1
Becker et al. (2018)	$F\left(S\right)=Q\left(\overset{M}{\underset{m=0}{\sum }}a_{m}\Sigma^{m}\right)Q^{T}$	*M*+1+(*N*^2^−*N*)/2
Deslauriers-Gauthier et al. (2020)	$F\left(S\right)=Q\left(\overset{M}{\underset{m=0}{\sum }}a_{m}\Sigma^{m}\right)Q^{T}+C$	*M*+1+*N*^2^

*For mappings whose input is L, the Laplacian of the structural connectivity matrix is computed before fitting the model. The diagonal matrix Σ contains the eigenvalues of the structural connectivity matrix S*.

### 2.6. Data and Data Processing

The data was provided by the Human Connectome Project^2^ (HCP) and our processing started from the minimally processed data (Glasser et al., 2013) for both diffusion and functional MRI data. The rest of pipeline is very similar to the one used in our previous work (Deslauriers-Gauthier et al., 2020), which we describe here again for completeness. For 100 unrelated subjects provided by the HCP, the brain was extracted using FSL bet (Smith, 2002) and the white matter, gray matter, and cerebrospinal fluid were segmented using FSL fast (Zhang et al., 2001). Using the FreeSurfer^3^ segmentation (Fischl, 2012) and the diffusion weighted images, a single fiber response function was computed for the white matter, gray matter, and cerebrospinal fluid using MRtrix3^4^ (Tournier et al., 2019). These response function were then used to compute a fiber orientation distribution function for each voxel using contrained spherical deconvolution (Tournier et al., 2007). Five million streamlines were generated using anatomically constrained probabilistic tractography using a step of 0.3 mm, a maximum length of 400 mm, and backtracking (Smith et al., 2012). In an effort to make connectomes more quantitative and to reduce the impact of false positive connections (Maier-Hein et al., 2017) by re-establishing the biological interpretability of streamline-based structural connections, the tractograms were filtered using SIFT2 (Smith et al., 2015) assigning a weight to each streamline representing its cross-sectional area. The cortical surface extracted with FreeSurfer was parcelated using the Schaefer atlas (Schaefer et al., 2018) into *N* = 200 regions. Given the parcellation and the streamlines, the structural connectome was built by summing the SIFT2 weights of streamlines connecting two cortical regions. The connectomes were symmetrized by summing the (*i, j*) and (*j, i*) entries of the connectome. Finally, the structural connectomes were normalized by dividing by the sum of the off diagonal entries and the diagonal set to zeros. While some authors removed weak connections we followed the recommendations of Civier et al. (2019) and omitted pruning thus keeping weak connections. The rational is that weak connections have little impact on values derived from connectivity matrices. It is therefore preferable to simplify the processing pipeline and reduce the number of arbitrary parameters by omitting this step.

The resting state functional MRI data provided by the HCP offers two sessions each containing two acquisitions, one with left to right encoding and one with right to left encoding. Here, we made use of the first (REST1) session and preprocessed the two acquisitions as follows. The time series were filtered using a butterworth bandpass filter with critical frequencies 0.01 and 0.1 Hz (van den Heuvel and Hulshoff Pol, 2010). The first 20 volumes of each acquisition were dropped and the remaining volumes linearly detrended (Caballero-Gaudes and Reynolds, 2017). The movement parameters and their derivatives were regressed out and the data was motion scrubbed (Power et al., 2012). After this preprocessing, we concatenated both acquisitions to produce a single dataset per subject. The mean signal for each parcel was computed and used to build the functional connectivity matrix using Pearson correlation. To preserve the SPD nature of the functional connectomes, the negative entries of the matrices were preserved, in contrast to our previous work.

## 3. Results and Discussion

### 3.1. Riemannian vs. Euclidean Distances

**Figure 1:**
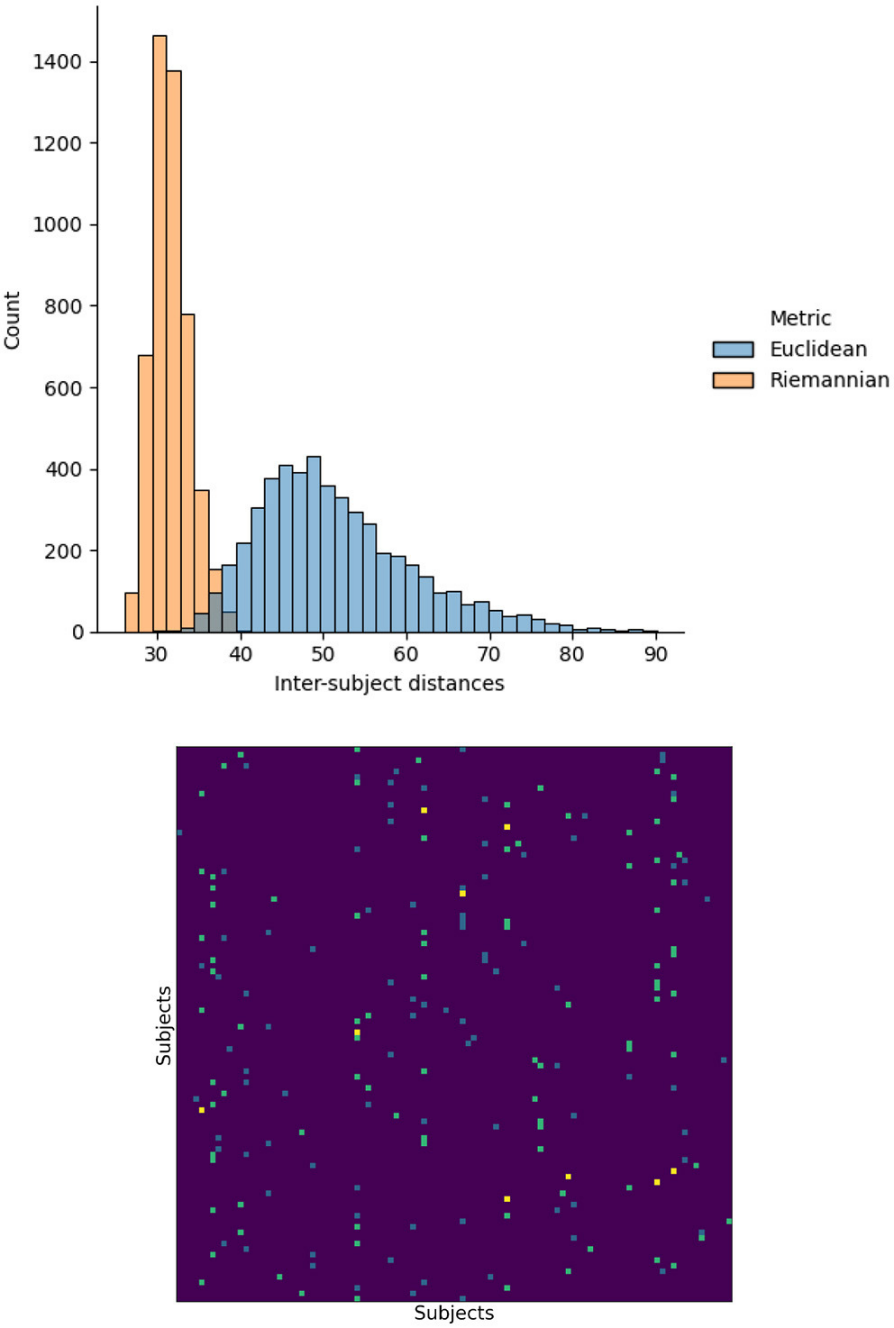
Histogram of the distances between the functional matrices of every subject using the Euclidean and Riemannian metrics. The nearest neighbor, minimizing each distance, are also presented. Teal and blue elements correspond to Euclidean and Riemannian nearest neighbors respectively and yellow indicates agreement between the two metrics.

First, we investigated the impact of measuring distances between functional matrices using the Euclidean or Riemannian metrics. To this end, we measured the distance between the functional matrices for every pair of subjects using both metrics. In addition, for each subject, we identified the other subject having the closest functional matrix, i.e., the nearest neighbor, again for both distances. The resulting distance and nearest neighbor matrices are illustrated in Figure 1. The Riemannian distances have a mean of 31.5∓2.3 with a range of 26.0 to 40.4 and the Euclidean distribution has a mean of 51.4∓9.3 with a range of 30.3 to 90.1. This change in distribution indicates that using the Riemannian distance significantly changes the relationship between functional matrices. To further confirm this observation, we note that the nearest neighbors for each distances do not agree. Indeed, only nine subjects had the same nearest neighbors using both distances, while 91 subjects differed. From these results, we conclude that the notion of similarity between subjects is significantly altered by the change in metric. Consequently, the relationship between structure and function will also be affect and reflected in the structure–function mappings.

**Figure 2:**
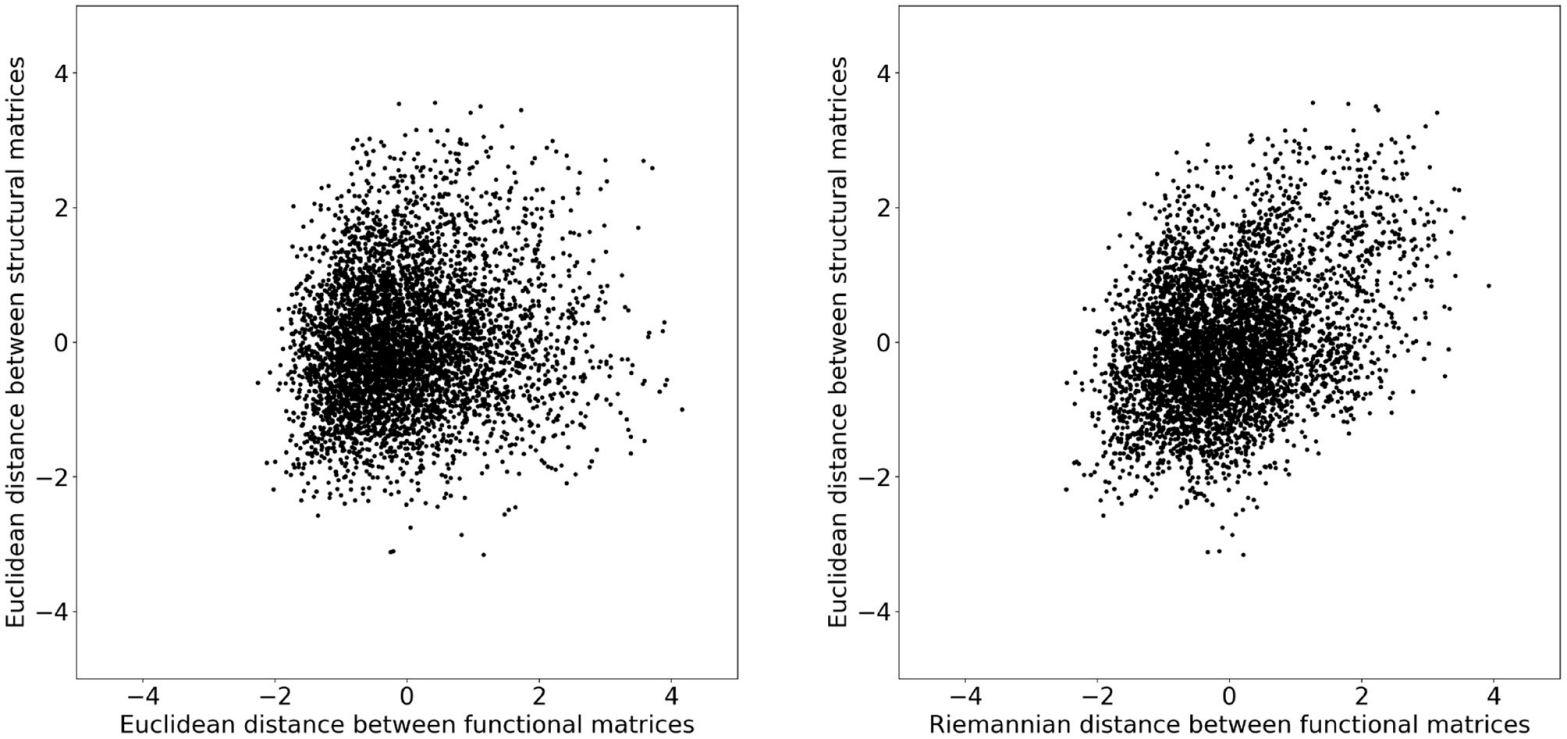
Distances between the structural matrices as a function of the distance between functional matrices for every subject. Both distances where converted to z-scores before plotting. On the **(Left)** plot, the distance between functional matrices is measured using the Euclidean metric and on the **(Right)** with the Riemannian metric. The coefficients of determination *R*^2^ are 0.016 and 0.135 for the Euclidean and Riemannian distances, respectively.

To investigate whether these changes improved the structure–function relation, we additionally computed the distance between structural matrices of every subject. Figure 2 illustrates the functional distance as a function of the structural distance for every subject. Both distances where converted to z-scores before plotting. We can observe that subjects that have a short structural distance are more likely to have a short functional distance when measured with the Riemannian distance rather than the Euclidean distance. To quantify this observation, we computed the coefficient of determination *R*^2^ for each distance and obtained 0.016 and 0.135 for the Euclidean and Riemannian distances, respectively. In other words, subjects that have a similar structural matrix also have a similar functional matrix, but only when distances are measured using the Riemannian metric. Note that these results are independent of any mapping strategy and instead provide evidence in favor of Riemannian metric on a more fundamental level. It is indeed expected that subjects with a similar structure should also have a similar function, but the *R*^2^ score near zero obtained by the Euclidean metrics would instead indicate a decorrelation between these two views of the brain.

To leverage this correlation between the structural and functional distances, we devised an additional mapping and included it in the following experiments. In this new mapping, the predicted functional connectivity matrix is given the Riemannian mean of the P training subjects whose structural connectivity matrices are closest to that the of the new subject, i.e., the P nearest structural neighbors. The rationale is that subjects having a similar structure will also have a similar function and averaging only the structural nearest neighbors will lead to an improve functional estimation. In the following section we refer to this mapping as the Riemannian nearest neighbors mean and empirically choose *P* = 50.

### 3.2. Structure–Function Mapping

**Figure 3:**
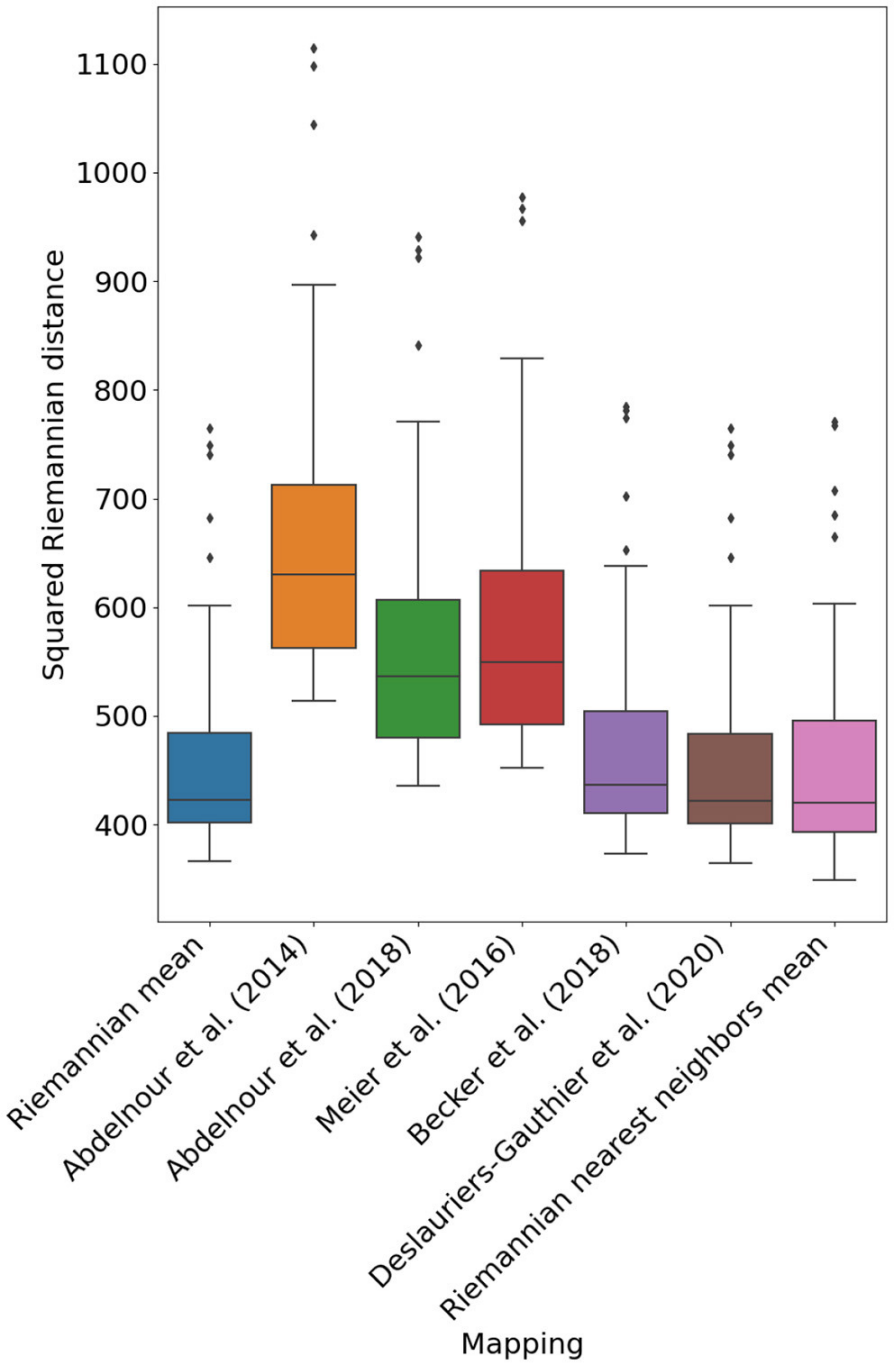
Mean squared Riemannian distance of five previously proposed mapping based on eigenmodes, the reference mapping (Riemannian mean), and the Riemannian nearest neighbors mean (*P* = 50).

Figure 3 illustrates the performance of the different mappings optimized using the procedure in Section 2.4. Notably, the mapping devised in Deslauriers-Gauthier et al. (2020) by adding a constant to the spectral mapping of Becker et al. (2018) obtains a mean squared distance (MSD) of 449.9 and outperforms the Riemannian mean which obtained a distance of 450.5. While the performance gain is admittedly small, we found the differences in errors to be statistically significant (*p* < 0.001). Another notable change is the inversion of the performance between the diffusion model of Abdelnour et al. (2018) and the spectral model of Meier et al. (2016) with respect to our previous study (Deslauriers-Gauthier et al., 2020). Using the Euclidean metric, the diffusion model was outperformed by the spectral model, a result that was justified by the parsimonious nature of the diffusion model. Using the Riemannian metric, the diffusion model obtained an MSD of 558.7 whereas the spectral model obtained 578.4. Except for this change, the performance relationship between previously proposed mapping strategies followed the same trends as in our previous work. In general, mappings with more degrees of freedom (see Table 1) provided better predictions, but at the cost of increased complexity. Finally, the Riemannian nearest neighbors mean mapping obtained the best performance overall with a MSD of 448.9. This improvement in performance illustrates that the correlation between structural and functional distances, only observed using the Riemannian metric, can be leveraged to improve our prediction of function from structure.

### 3.3. Limitations

One of the fundamental assumptions in structure–function mapping is that changes in the structural connectivity of subjects affects their functional connectivity. However, this study considered healthy young adults, a very homogeneous cohort with similar structural connectivity. As such, the structural differences between subjects are subtle and may contribute to the high prediction accuracy of the mean. A second limitation is the use of existing mappings that were not explicitly designed with the tools of Riemannian geometry. This may also contribute to the high performance of the mean, which is optimal in a Riemannian sense. An interesting avenue of research is the design of mappings that leverage the topology of the space of SPD matrices to improve the predictions performance. A simple proof-of-concept for this approach is the Riemannian nearest neighbors mean which obtained the lowest MSD, and thus the best functional prediction.

It is also important to note that the impact of the different processing steps on our ability to predict function from structure in a Riemannian setting was not evaluated. Indeed, both the functional and structural connectivity pipelines require the selection of parameters which will modify the estimated connectivity. For example, structural matrices can be constructed by counting streamlines or by using a weighting strategy such as COMMIT (Schiavi et al., 2020) or SIFT2 as was done here. To investigate the importance of this specific choice, we reproduced the experiments using streamline counting structural connectomes. The results obtained (available in Supplementary Materials), are close to those produced by SIFT2 connectomes and do not alter the conclusions. However, this may not be the case for every step of the pipelines and an exhaustive search may lead to further improvement of the predictions.

Finally, the structural connectivity matrices of this study only considered white matter cortico-cortical connections. Other connections, for example those including subcortical nodes and superficial white matter fibers (Reveley et al., 2015), are not encoded in the matrices. As such, it is unlikely that functional connectivity can be completely predicted by the structural matrices. Adding these missing connections may very well improve the performance of structure-function mappings, in addition to providing a more complete view of structural connectivity.

## 4. Conclusions

In this work, we investigated the implications of using a distance based on an affine invariant Riemannian metric in the context of structure–function mapping. We argued that functional matrices, if they are not thresholded, are symmetric positive definite and therefore live in a convex half–cone in the vector space of symmetric matrices. By using a distance appropriate to this manifold, we showed that a previously proposed mapping is able to outperform the group average, a result which was not obtained with an Euclidean metric. While the improvement in performance with respect to the mean was moderate, it should be observed that the mappings were not specifically designed with this manifold in mind and could thus be improved. In addition, while we chose to focus on mappings based on eigenmodes, our findings may also have interesting extensions to deep learning approaches for structure-function mapping (Ji et al., 2021). Indeed, the already promising performance of these approaches may be further enhanced by the use of Riemannian geometry. In conclusion, our results show that using the correct distance for SPD matrices improved the performance of existing structure–function mappings and may lead to better prediction with more specific models.

## Data Availability Statement

Publicly available datasets were analyzed in this study. This data can be found here: Human Connectome Project (HCP) Young Adult, https://db.humanconnectome.org.

## Author Contributions

SD-G: conceptualization, methodology, software, investigation, writing—original draft, and visualization. MZ: conceptualization, methodology, and writing—review and editing. HL: methodology and writing—review and editing. RD: conceptualization, methodology, writing—review and editing, supervision, and funding acquisition. All authors contributed to the article and approved the submitted version.

## Funding

This work has received funding from the European Research Council (ERC) under the European Union's Horizon 2020 research and innovation program (ERC Advanced Grant agreement no. 694665: CoBCoM—Computational Brain Connectivity Mapping). This work has been supported by the French government, through the 3IA Côte d'Azur Investments in the Future project managed by the National Research Agency (ANR) with the reference number ANR-19-P3IA-0002. The authors are grateful to the OPAL infrastructure from Université Côte d'Azur for providing resources and support.

## Conflict of Interest

The authors declare that the research was conducted in the absence of any commercial or financial relationships that could be construed as a potential conflict of interest.

## Publisher's Note

All claims expressed in this article are solely those of the authors and do not necessarily represent those of their affiliated organizations, or those of the publisher, the editors and the reviewers. Any product that may be evaluated in this article, or claim that may be made by its manufacturer, is not guaranteed or endorsed by the publisher.

## Footnotes

^1^ https://github.com/pymanopt/pymanopt

^2^ https://www.humanconnectome.org

^3^ https://surfer.nmr.mgh.harvard.edu/

^4^ http://www.mrtrix.org
