# Supplementary material for: A Riemannian Revisiting of Structure–Function Mapping Based on Eigenmodes
Source: Front Neuroimaging. 2022 May 25;1:850266. doi: 10.3389/fnimg.2022.850266 (PMC10406294; doi:10.3389/fnimg.2022.850266)
Supplement: Supplementary file 1 [file Data_Sheet_1.PDF]

Supplementary Material to
A Riemannian revisiting of structure–function mapping based on eigenmodes

Samuel Deslauriers-Gauthier, Mauro Zucchelli, Hiba Laghrissi, and Rachid Deriche

Center Inria d'Université Côte d'Azur, France

1 Mapping using streamline count matrices

In the following, we reproduced the experiments leading to Figures 2 and 3 of the mansucript, but using an alternate structural connectivity matrix. Instead of quantifying the structural connecvity using the sum of the weights produced by the SIFT2 algorithm, we used the number of streamlines connecting each region. All other steps of the experiments are identical.

For this altered methodology, the distances between the structural matrices as a function of the distance between functional matrices (Figure 2 in the manuscript, Figure 1 here) exhibit the sames features as SIFT2 connectomes. In particular, a correlation between the distances is only observed when using a Riemannian distance. Likewise, the performance of all mappings remained almost identical when using the count matrices instead of the SIFT2 matrices (Figure 3 in the manuscript). Overall, these results are in agreement with our previous work (Deslauriers-Gauthier et al., 2020).

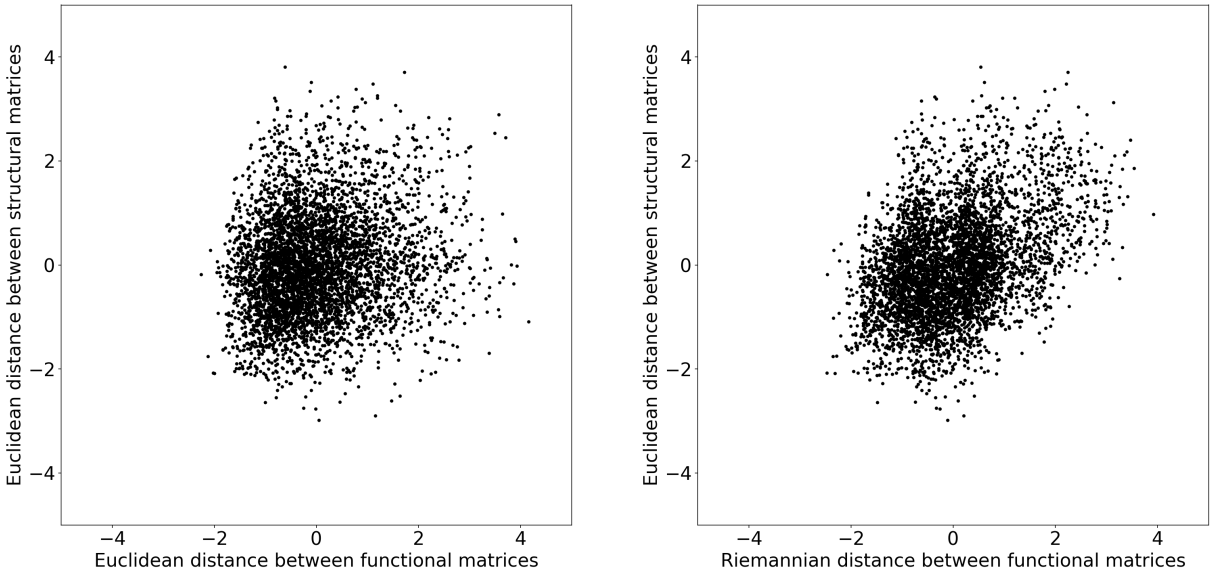

Figure 1: Distances between the structural matrices (streamline count) as a function of the distance between functional matrices for every subject. On the left plot, the distance is measured using the Euclidean metric and on the right with the Riemannian metric.

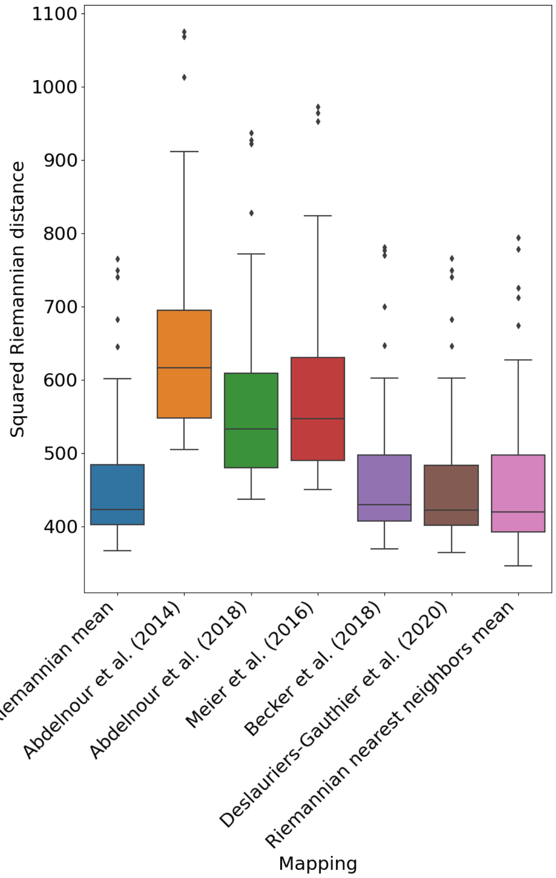

Figure 2: Distances between the structural matrices (streamline count) as a function of the distance between functional matrices for every subject. On the left plot, the distance is measured using the Euclidean metric and on the right with the Riemannian metric.
